# A multi-scale approach to describe electrical impulses propagating along actin filaments in both intracellular and *in vitro* conditions†
†Electronic supplementary information (ESI) available. See DOI: 10.1039/c7ra12799e


**DOI:** 10.1039/c7ra12799e

**Published:** 2018-03-28

**Authors:** Christian Hunley, Diego Uribe, Marcelo Marucho

**Affiliations:** a Department of Physics and Astronomy, The University of Texas at San Antonio, San Antonio, TX 78249-5003, USA. Email: marcelo.marucho@utsa.edu

## Abstract

An accurate and efficient characterization of the polyelectrolyte properties for cytoskeleton filaments are key to the molecular understanding of electrical signal propagation, bundle and network formation, as well as their potential nanotechnological applications. In this article, we introduce an innovative multi-scale approach able to account for the atomistic details of a protein molecular structure, its biological environment, and their impact on electrical impulses propagating along wild type actin filaments. The formulation includes non-trivial contributions to the ionic electrical conductivity and capacitance coming from the diffuse part of the electrical double layer of G-actins. We utilize this monomer characterization in a non-linear inhomogeneous transmission line prototype model to account for the monomer–monomer interactions, dissipation and damping perturbations along the filament length. A novel, simple, accurate, approximate analytic expression has been obtained for the transmission line model. Our results reveal the propagation of electrical signal impulses in the form of solitons for the range of voltage stimulus and electrolyte solutions typically present for intracellular and *in vitro* conditions. The approach predicts a lower electrical conductivity with higher linear capacitance and non-linear accumulation of charge for intracellular conditions. Our results show a significant influence of the voltage input on the electrical impulse shape, attenuation and kern propagation velocity. The filament is able to sustain the soliton propagation at almost constant kern velocity for the *in vitro* condition, whereas the intracellular condition displays a remarkable deceleration. Additionally, the solitons are narrower and travel faster at higher voltage input. As a unique feature, this multi-scale theory is able to account for molecular structure conformation (mutation) and biological environment (protonations/deprotonations) changes often present in pathological conditions. It is also applicable to other highly charged rod-like polyelectrolytes with relevance in biomedicine and biophysics.

## Abstract 2

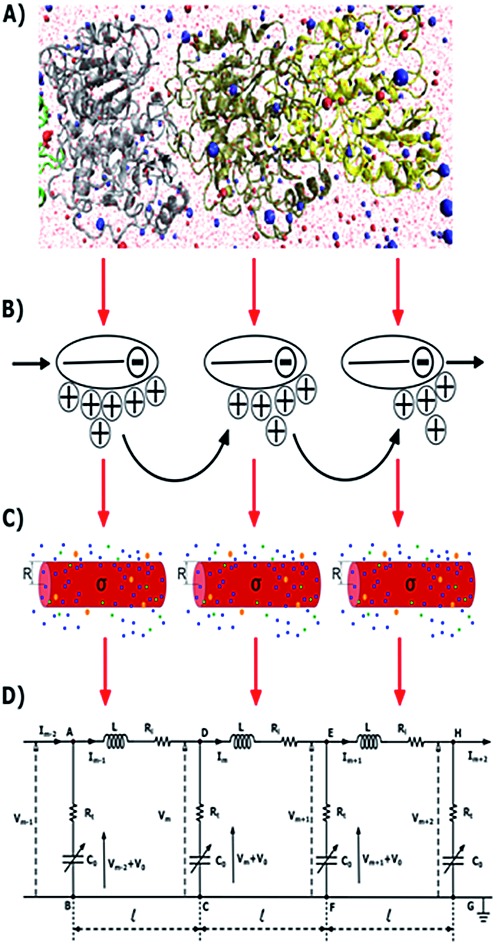
An innovative analytic solution accounting for the molecular structure, its biological environment, and their impact on electrical impulses along microfilaments.

## 1. Introduction

Actin filaments (F-actin) are long charged rod-like cytoskeleton polymers, which carry out many important biological activities in eukaryotic cells.^1^,^2^ These microfilaments have recently gained wide notoriety for their fascinating polyelectrolyte properties.^3^ According to single filament experiments in solution,^4^,^5^ F-actin have been shown to sustain ionic conductance and transmit electrical currents in the form of localized counterionic waves about the polymer's surface. The velocity of propagation along the surface of an actin microfilament is consistent with the velocity of propagation for neuronal impulses. Hence, in principle, concurrent propagation of both electrical signals along actin microfilaments, and electrochemical currents along the axonal membrane are highly possible. Another intriguing property of these proteins is their capacity for overcoming electrostatic interactions to form higher-order structures (bundles and networks) in the cytoplasm. For instance, cytoskeletal filaments are often directly connected with both ionotropic and metabotropic types of membrane embedded receptors, thereby linking synaptic inputs to intracellular functions.^6^ Conducting microfilaments may also govern at least some aspects of overall ion channel behavior within microvilli.^7^,^8^

All of these observations provide strong evidence on the polyelectrolyte nature of F-actin, which provides unique, yet still poorly understood, conducting and bundling formation properties in a variety of neuron activities including intracellular information processing, regulating developmental plasticity, and mediating transport. Certainly, the molecular understanding of the polyelectrolyte properties of cytoskeleton filaments will not only open unexplored frontiers in biology and biomedicine, but it will also be crucial for the development of reliable, highly functioning small devices with biotechnological applications such as bionanosensors and computing bionanoprocessors.^9^–^13^ Therefore, it is of crucial importance to determine the underlying biophysical principles and molecular mechanisms that support the ionic conductance and electrical impulse transmission in actin filaments under a variety of biological environments.

**Fig. 1:**
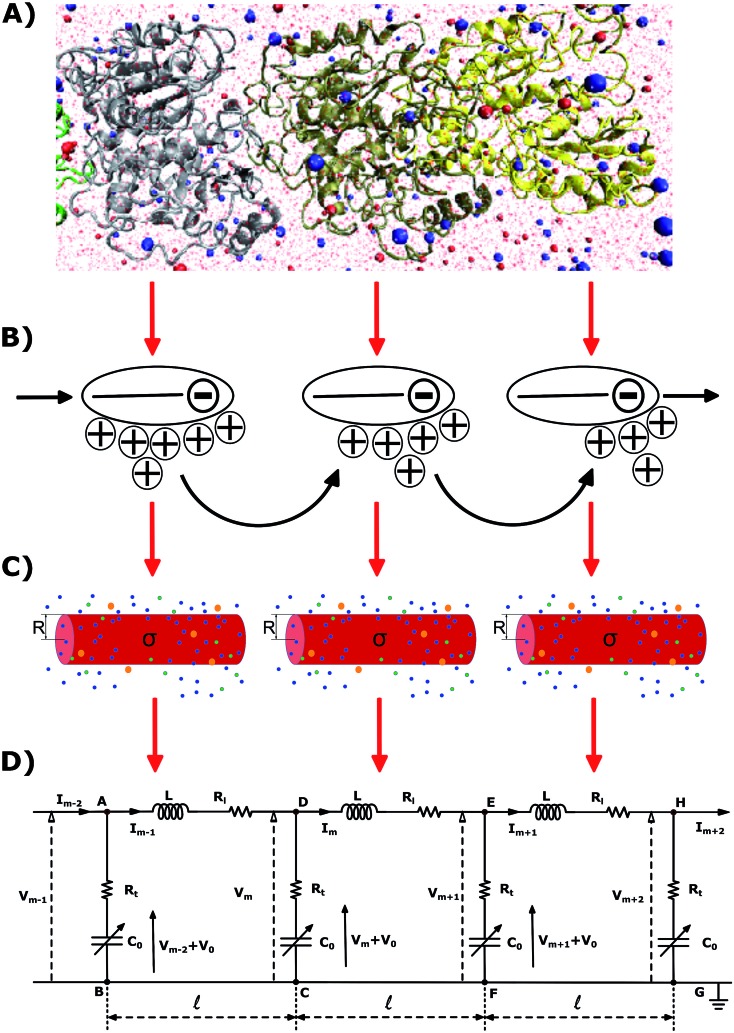
(A) Molecular structure model. (B) Ion condensation theory. (C) Cylindrical biomolecule model (D) Dispersive transmission line model.

The current understanding of these phenomena builds on a pioneers work. Fumio Oosawa suggested around 50 years ago that electric signals could be channeled through a medium along a microfilament due to the electrolyte solution forming an electron cloud along the filament length.^14^ It was Manning who introduced condensation theory,^15^ which provided the foundation for linear polymers to enable electrical currents in the form of ionic movements. As charged polyelectrolytes, cytoskeleton filaments may contain a proportion of their surrounding counterions in the form of a dense or “condensed” cloud about their surface, as long as, there is a sufficiently high linear charge density, a critical concentration of multivalent ions, and a small dielectric constant of the surrounding medium. These criteria are indeed met for actin filaments in neurons.^14^,^16^,^17^ Further, molecular structure analysis indicates the distribution of counterion clouds is non-uniform along the filament's length. This is due to F-actin originating from the linear polymerization of globular actin (G-actin) units. Each of these units have tight binding sites that mediate head-to-tail interactions to form a double-stranded helix. Therefore, resembling a solenoid with a fluctuating current flowing as a result of voltage differences generated by the ends of the filament. Additionally, with the filament core separated from the rest of the ions in the bulk solution by the counterion condensation cloud, this overcast of counterions may act as a dielectric medium between the filament and bulk layer. Hence, providing F-actin both resistive and capacitive behaviors that may be associated with a highly conductive medium. This conduction along microfilaments is characterized by the decomposition of an electrical input pulse into discrete delayed charge portions. These patterns clearly indicate the existence of charge centers with corresponding counter ion clouds along the polymer axis. As a consequence, electrically forced ions entering one end of the biopolymer will result in ions exiting the other end (Fig. 1). Therefore, actin polymers may serve as “electrical nanobiowires” whom can be modeled as non-linear inhomogeneous transmission lines known to propagate non-linear dispersive solitary waves.^18^ These waves can take the form of localized electrical signal impulses.^19^–^21^ However, this basic understanding about the electrical impulses propagating along actin filaments does not account for all conductance properties of microfilament bundles.^22^ More recent approaches, based on Gouy-Chapman electrical double layer type models and mean-field Poisson-Boltzmann (PB) theories, provide further insight into the ionic equilibrium distributions and electric potential properties near the polymer surface, which arise from the charged polyelectrolyte surface, continuum solvent dielectric medium, and mean electrostatic potential energy generated by mixed salts comprised of point-like ions.^23^–^27^ These methods are shown to break down for cytoskeleton filaments under certain situations, because they entail several approximations in their treatment of the ions and solvent molecules. They don't account for water crowding, ion size asymmetry or electrostatic ion correlation effects, all of which are likely to play a fundamental role in providing a quantitative description of the polyelectrolyte nature of cytoskeleton filaments, and consequently, their conducting and electrical signal propagation properties.^28^–^31^

In this article, we introduce a novel multi-scale (atomic (Å) → monomer (nm) → filament (μm)) approach to describe non-linear dispersive electrical impulses propagating along actin filaments (see Fig. 1). In section II, an atomistic model of F-actin^32^ and its biological environment is used to determine the polyelectrolyte properties and molecular mechanisms governing G-actins in the polymerization state. This approach, along with a suitable modification of Nernst–Planck theory,^33^ are used to calculate the monomeric radial and axial flow resistances. However, a more sophisticated approach based on a classical solvation density functional theory^31^,^34^–^37^ is required to calculate the monomeric radial ionic capacitance. In section III, we extend the approach to capture the biophysics and biochemistry at larger (microfilament) scale distances. We utilize the monomer characterization in a non-linear inhomogeneous transmission line prototype model to account for the monomer–monomer interactions, and ergo, the electrical impulse propagation along the filament length. A novel approximate analytic solution is obtained for this model and utilized in section IV to characterize the electrical impulse peak, width, and velocity of propagation under several voltage stimulus and electrolyte conditions.

## 2. G-actin characterization in the polymerization state

### 2.1. Cylindrical biomolecule model for G-actins

**Fig. 2:**
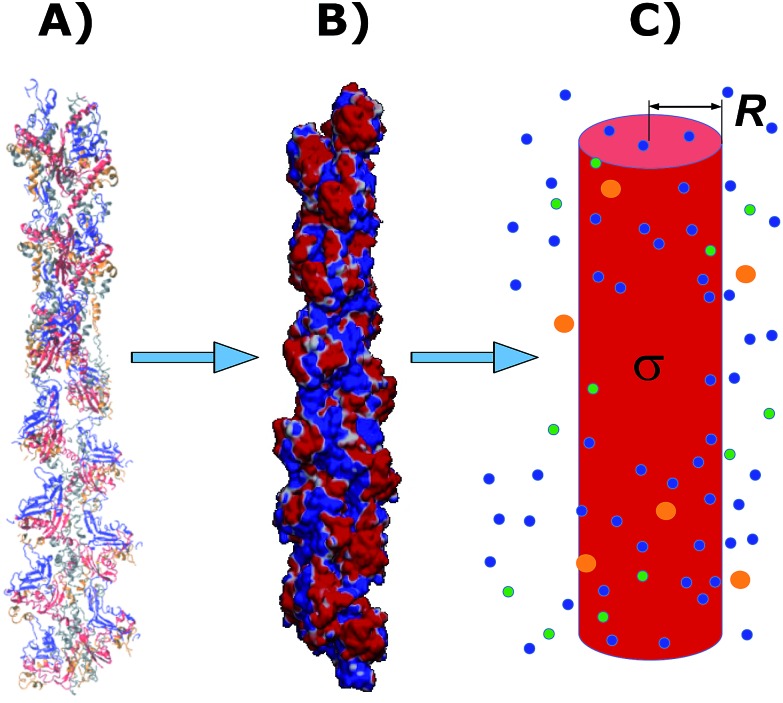
A Cong model representation of F-actin used to visualize the polymerized protein from three different viewpoints when immersed in a saline solution of sodium chloride at pH 7.2. (A) A ribbon diagram of 13 monomers polymerized into a single actin filament illustrating the effects of the electrolyte on the residues exposed to the surface. (B) A filament volume representation of F-actin showing potential on the surface of the actin filament (C) a cylindrical characterization of F-actin with radius *R* and surface charge density *σ*, used to calculate the components of the electric circuit model (see Fig. 3), with surrounding ions.

The physicochemical properties of each monomer composing the actin filament are different from those as single globular actin proteins, because polymerization into filamentous form generates several conformational changes to each monomer. Therefore, we retrieve the information for the monomers molecular structure by using one of the most recent 13 monomer, biologically assembled, wild type F-actin filament models posted on the protein data bank: the Cong model^32^ (see Fig. 2a). It provides a detailed molecular characterization including the amino acid sequence and the number and type of residues exposed to the electrolyte. This uncharged molecular structure in pdb format is uploaded into pdb2pqr webserver^38^ to assign atomic charges and sizes, add hydrogens, optimize the hydrogen bonding network, and renormalize atomic charges of the residues exposed to the surface due to pH effects (protonation/deprotonation process). The resulting charged molecular structure at pH = 7.2 is used to extract information on the effective filament length *L* = *Z*_max_ – *Z*_min_ = 422.20 Å and monomer length *𝓁* = *L*/13 = 5.4 nm, as well as the total filament charge *Q* = ∑*q*_i_ = –154*e*, where *e* is the electronic charge (see Fig. 2b). As a first approximation, the filament length and total charge are used to estimate the filaments linear charge density 
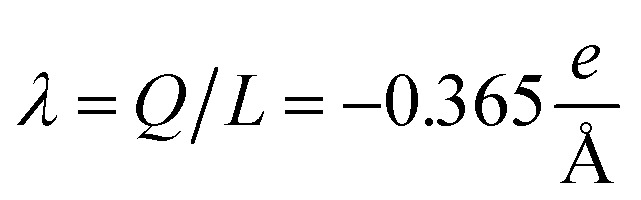
. Additionally, the molecular structure is uploaded into ‘‘3v: voss volume voxelator” webserver^39^ to estimate the total filament volume *V*_p_ = 753 006 Å^3^. From here the effective monomer radius *R* of the molecular structure model is calculated 
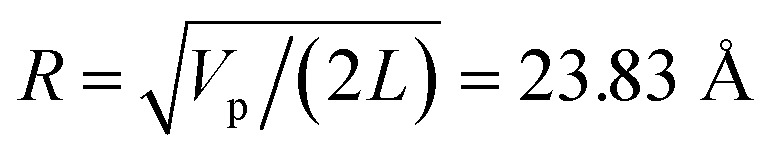
. The linear charge density and radius are subsequently used to calculate the filament (=monomer) surface charge density 
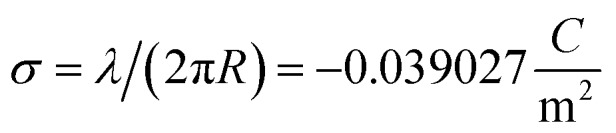
.

**Fig. 3:**
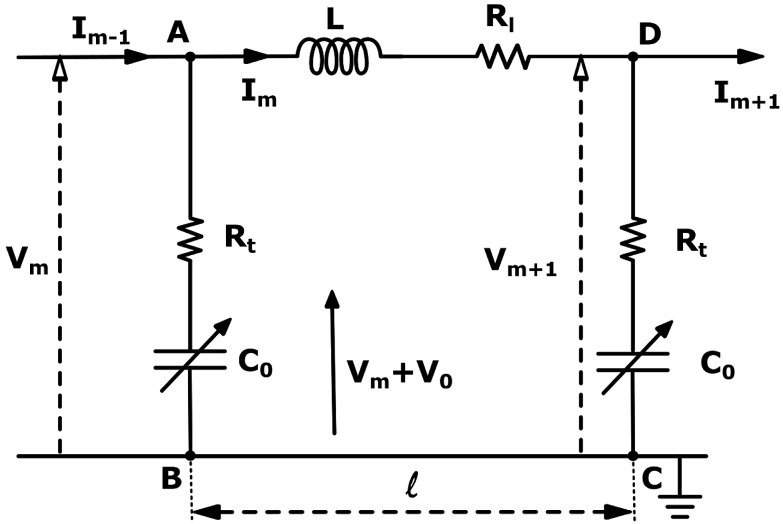
An Effective circuit diagram for the *m*^th^ monomer of a chain of monomers which make up an actin filament with four nodes labeled A, B, C, D. The monomers in the sequence are denoted by *m*, where *m* – 1 and *m* + 1 are the preceding and succeeding adjacent monomers, respectively. The potential across monomer *m* is indicated by *V*_*m*_, where *V*_0_ is the constant DC bias electric potential, and *𝓁* is the monomer length. The components of the circuit are the longitudinal and transversal resistance *R*_l_ and *R*_t_, respectively, *C* is the capacitance, and *L* the self inductance.

It is worth mentioning that axial and azimuthal symmetry assumptions often used for rod-like polyelectrolytes have been shown to capture important features of the electrical double layer (EDL) properties without computational restrictions, mainly, for low electrolyte concentrations and filaments with an attribute of length much larger than its effective radius.

In the next sections we use the effective radius *R*, length *𝓁* and surface charge density *σ* to determine the longitudinal and transversal ionic flow resistances, capacitance and self-inductance for each monomer along the filament (see Fig. 2c).

### 2.2. Electrical and conductive properties of G-actins in solutions

The electrical properties of a single G-actin are usually characterized by an electric circuit containing a capacitor, two resistances and a self-inductance component. The capacitor, whose capacitance changes with applied voltage, originates the non-linearity behavior of the electrical impulse. The structural periodicity in the arrangement of monomers generates the dispersion of the electrical impulse along the filament. Whereas, the losses in the transmission media is accounted for by a longitudinal (series) and transversal (shunt) resistors, which represent the finite conductivity of the conductors and the dielectric insulator between the conductors, respectively. Additionally, the inductive components contribution to the electrical properties of the electrical impulse is due to F-actin's double-stranded helical structure, which induces the ionic flow in a solenoidal manner around each monomer. Clearly, these phenomena depend on the monomeric physicochemical properties. In this work, we use the cylindrical biomolecule model for G-actins described in the previous section. A detailed calculation on the electrical and conductive properties of G-actins in solutions is provided in the ESI† document.

We use a novel approach by combining transport and Ohm's laws, as well as, Navier–Stokes and Poisson's theories to obtain simple and accurate, approximate analytic expressions for the radial (transversal) *R*_t_ and axial (longitudinal) *R*_l_ ionic flow resistances. We assume that the radial electrolyte convection is neglectable. For long filaments and uniform monomer surface charge densities, we also assume that the charged ions are distributed radially around the biomolecule, which in turn, originates an azimuthal and axial symmetry on the resulting electric potential. By solving the linearized PB equation, we obtain the following approximate expression for the mean electrostatic potential1
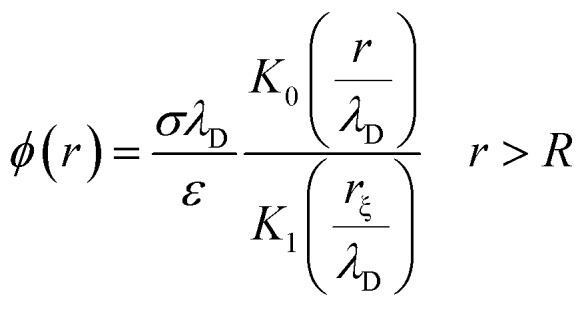
where *K* is the modified Bessel function of the second kind, *λ*_D_ represents the Debye length (electrical double layer width estimation), *σ* stands for the monomer surface charge density, and *r*_ξ_ ≃ *R* is the radius predicted by the cylindrical model. This approximate solution deviates no more than 9% from the exact NLPB equation solution and recovers the result of previous work^24^ in the case of 
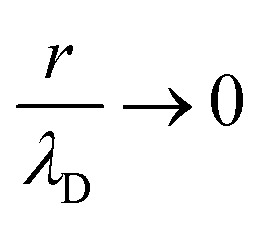
.

For the longitudinal ionic flow resistance calculations, we consider a voltage drop Δ*V* between the monomer ends, which generates a uniform axial electric field along the monomer 
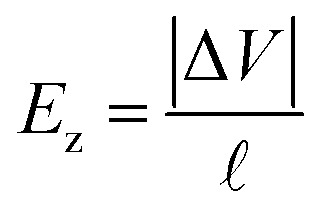
, and consequently, a electro-osmotic (migration) force on the ions in the electrolyte. We also consider the natural convection force to account for the axial velocity profile *v*_z_(*r*). This is omitted in previous work,^24^ however, it plays an important role in characterizing the movement of the fluid near the protein surface. After some algebra and analytic calculations, the following expression for the longitudinal ionic flow resistance is obtained2
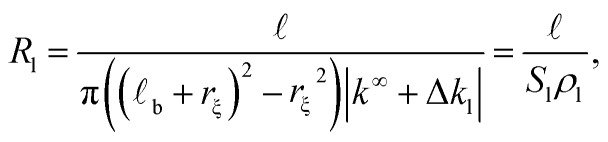
where *S*_l_ = π((*𝓁*_b_ + *r*_ξ_)^2^ – *r*_ξ_^2^) represents the effective cross section surface area facing perpendicular to the longitudinal ionic flow, *r*_ξ_ ≃ *R* is the slip velocity position, *𝓁*_b_ stands for the Bjerrum length, *ρ*_l_ = |*k*^∞^ + Δ*k*_l_| is the effective axial ionic conductivity, and *k*^∞^ is the bulk electrolyte conductivity. Whereas, Δ*k*_l_ accounts for those contributions to the radial conductivity coming from the diffuse part of the electrical double layers formed by the electrolyte around G-actins.

A similar approach is used for the transversal ionic flow resistance calculations. Here we assume the electro-osmosis generated by the gradient of the radial electric potential as the only driving (migration) force dominating the radial surface current density. As a result, the radial ionic flow resistance is given by3
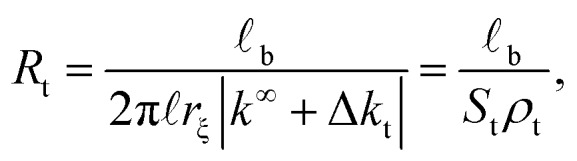



In the latter expression, *S*_t_ = 2π*𝓁r*_ξ_ represents the effective lateral surface area facing perpendicular to the radial ionic flow, *ρ*_t_ = |*k*^∞^ + Δ*k*_t_| is the effective transversal ionic conductivity, whereas, Δ*k*_t_ represents those contributions to the transversal conductivity coming from the diffuse part of the electrical double layers formed by the electrolyte around G-actins.

Expressions (2) and (3) show that our approach extends the capabilities of previous work for cytoskeleton filaments^24^,^25^ by accounting for non-trivial ionic conductivity contributions, namely Δ*k*_𝓁_ and Δ*k*_t_, which depend on the protein surface charge density, protein radius, Bjerrum length, and electrical double layer width. These contributions cannot be neglected in general (numerical values are provided below). Indeed, only when they are considered in the calculations, is it possible to provide a more accurate description of the conductivity properties for charged filaments.

It is worth mentioning that the mean-field theories utilized thus far for the previous calculations are justified under the conditions considered in this article, namely *𝓁*_b_ ⪅ *λ*_D_, and *d*/*λ*_D_ < 1, where *d* stands for the average ionic diameter. However, they are inaccurate, and consequently, inappropriate to describe the differential capacitance of electric double layers in ionic liquids.^28^,^29^ In these systems, the capacitance is not constant, but depends on the applied voltage due to the ion condensation around the protein surface. For small voltages, a linear relationship is usually considered, being that the proportionality constant is an unknown, free parameter of the theory. In the present article, we overcome this issue by using a more sophisticated approach, the so-called classical solvation density functional theory (CSDFT). The approach extends the capabilities of PB formalism by considering not only the electric, but also the entropic and many-body interactions. This feature has been particularly useful for identifying and characterizing dominant interactions and molecular mechanisms governing the behavior and function of macroions under a variety of electrolyte conditions.^34^–^37^ The approach, successfully tested on double stranded segments of B-DNAs in normal conditions,^34^ is expected to provide an accurate characterization of the polyelectrolyte properties of a segment of F-actin (*e.g.* a monomer), and consequently, the capacitance of its cylindrical electrical double layer (see Fig. 2) due to the strong similarities between these two rod-like biopolymers.^3^ Our calculations, based on the Cong molecular structure and the cylindrical biomolecule model for G-actins described above, yields the following expression for the total charge accumulated in the capacitor *Q* and monomer capacitance *C*(*V*)

4 *Q* = *C*_0_(*V* – *bV*^2^) = *VC*_0_(1 – *bV*) = *VC*(*V*)

where *C*_0_ represents the linear capacitance of the capacitor and *b* the theory's parameter characterizing the non-linear behavior (numerical values for these parameters are provided below).

The last component of the electric circuit, *e.g.* the self-inductance *L* for G-actins, is usually estimated using Faraday's law^24^5
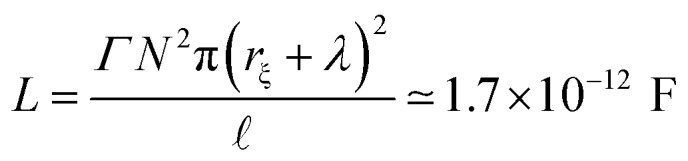
where *Γ* and *N* represent the magnetic permeability and the number of turns (*e.g.* how many ions could be lined up along the length of a monomer), respectively. Here *N* ≃ *d*/*𝓁*, where *d* is the average ion size. According to this rough estimation, the self-inductance contribution to the electrical signal propagation for F-actins is expected to be neglectable, however it may play an important role in other highly charged filaments. Therefore, the parameter will be considered in the present work as the approach may be useful for future applications.

## 3. Lossy, non-linear, dispersive transmission line model for microfilaments

Loan counterions surrounding microfilaments may be transferred from one charge center (*e.g.* monomer) to the next, giving rise to a locally restricted excess of partial charges that represent the propagation of electrical impulses along the conducting pathway.^8^ This kind of charge transfer mechanism between monomers is well known to generate flow of weakly bound electrons in conducting polymer systems where the electrical current and propagation velocity are different in nature. These transmission line models have been successfully demonstrated to characterize electron currents along conducting polymers, and are often utilized to describe ionic conductance and electrical impulse propagation along cytoskeleton filaments.^18^,^26^,^40^ In this article, we use the sequential arrangement of elementary electric units introduced in the pioneer work of Tuszyński & Cantiello,^24^ where each unit represents a single G-actin characterized by the capacitor, resistances and self-inductance described in the previous section. A detailed characterization of the non-linear, dispersive transmission line model is provided in the ESI† document.

Application of Kirchhoff's laws on the discrete transmission line model constructed by *N* elementary cells (monomers), along with relationship (4), lead to the following coupled differential equations for describing the electrical potential *V*_*m*_(*t*) = *Z*^1/2^*U*_*m*_(*t*) across a cell unit *m*6
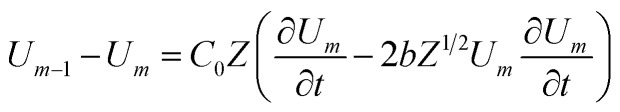

7
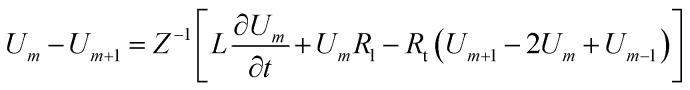
where *Z* represents the characteristic impedance of the cell unit (numerical values for this parameter are provided below). Since the individual monomer length *l* is much smaller than the actin filament length (*N* – 1)*l*, we can approximate eqn (6) and (7) as the voltage *U*_*m*_ and current *I*_*m*_ traveling down the actin filament by moving from one adjacent circuit modeled monomer to the next. Additionally, we can use the conventional continuum approximation for the electrical potential *U*_*m*_(*t*) ⋍ *U*(*x*, *t*), and a Taylor series in terms of the parameter *𝓁*, to get the following perturbated Korteweg-de Vries (pKdV) differential equation^21^,^41^ for the electric potential time evolution along a single F-actin8
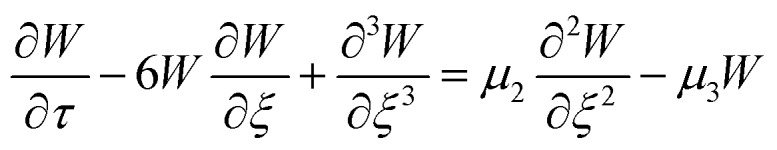



The latter equation is expressed in terms of dimensionless variables *ξ*(*x*, *t*), *τ*(*t*), re-scaled potential 
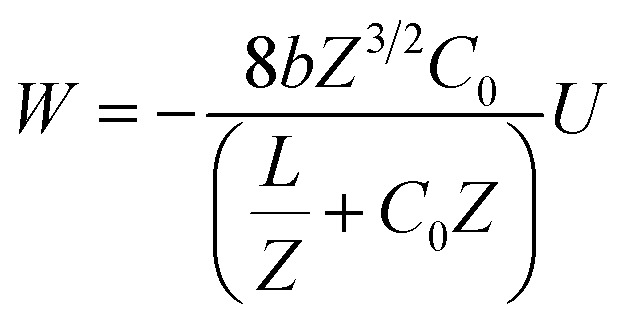
, and new parameters 
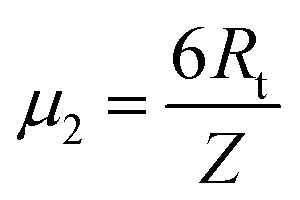
, 
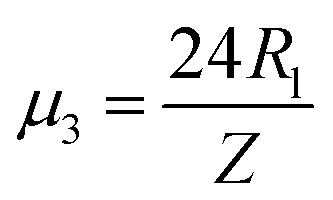
. The first term to the left resembles the time dependent term in Fick's diffusion law. The second and third terms represent the non-linearity and dispersive contributions arising from the condensed ion cloud in the electrical double layer and the diffuse spreading of ions along the microfilament, respectively. On the other hand, the first and second terms to the right represent the dissipation and damping perturbations, respectively.

Eqn (8) appears in a variety of systems^42^ describing the propagation of electrical solitons for a non-linear dispersive transmission line in the form of localized voltage waves. A similar equation was recently derived for microtubules,^26^ and for the first time, obtained here for actin filaments. For these transmission lines, an initial pulse *W* (*ξ*, 0) may decay into a sequence of solitons and a tail. As a first approximation, we will consider single soliton solutions in the framework of the perturbation theory on the basis of the adiabatic approximation.^43^ According to this approach, an external voltage input *V*_inp_ generates the propagation of a soliton pulse *W* (*ξ*, *τ*) in the following form

9 *W*(*ξ*, *τ*) = –2[*Ω*(*τ*)]^2^sech^2^[*Ω*(*τ*)(*ξ* – *η*(*τ*))]

where *Ω*(*τ*) and *η*(*τ*) are given by the expressions10
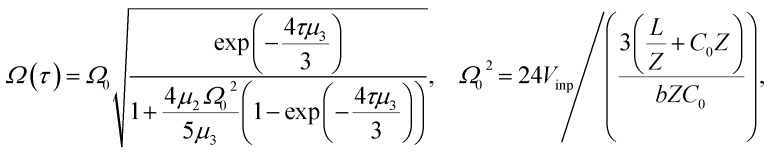
and11
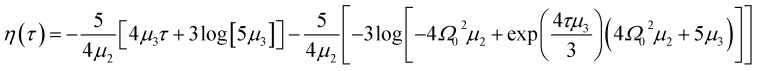



By rewriting the argument of solution (9) in terms of the original variables12
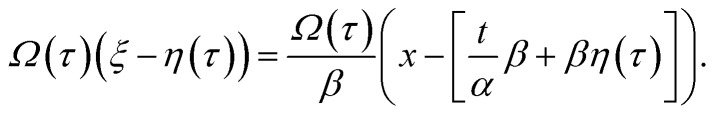
the soliton pulse *W*(*ξ*, *τ*) can be characterized by the time dependent wave number 
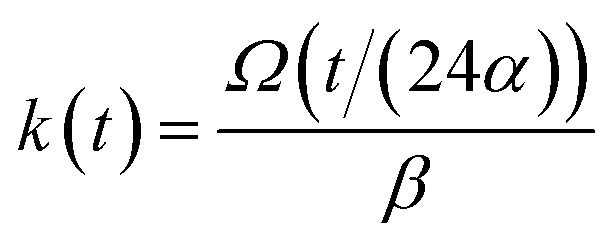
 and kern propagation velocity along the filament in units of m s^–1^13
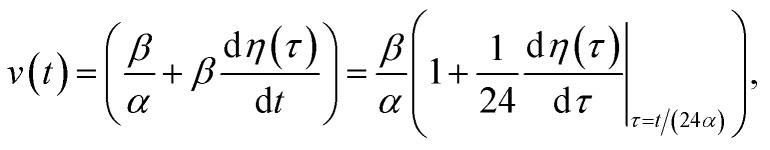
where14
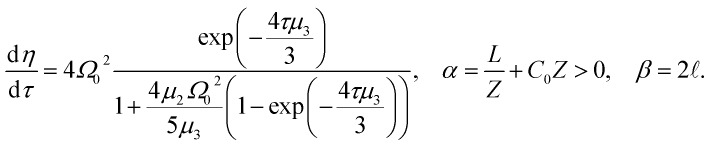



The time average kern velocity of the soliton in a time interval [0, *t*_max_] is given by the usual expression15
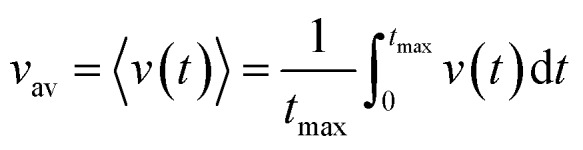



In the latter expression, *t*_max_ represents the vanishing time (or time width), which is considered in this work as the time taken by the initial soliton amplitude to be attenuated 99%, namely 
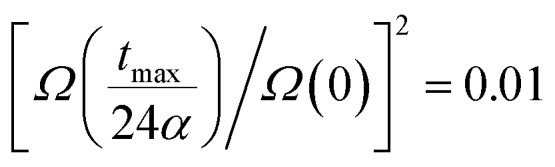
.

It is worth noting that the approximate analytic solution (9) was compared with the exact numerical solution of the differential eqn (8) for both intracellular and *in vitro* conditions, obtaining a good visual matching over the whole domain. Clearly, eqn (9) accounts for amplitude, shape and velocity attenuation when propagating due to the ionic flow resistivities, inductance, and capacitance characterizing the G-actin monomers. While a similar soliton pulse behavior was recently found for microtubules,^26^ previous work for actin filaments were looking for solitary-wave solutions that propagate without changing form.^24^ This may only be expected when a balance between non-linearity and dispersion occurs, which requires that the effect of the perturbing terms on the shape of the soliton cancel each other out. We note that this kind of particular soliton arise in our approach when the right side of eqn (8) is indeed neglected. Nevertheless, our results show that this cancellation does not occur for the conditions considered in the present work.

## 4. Results and discussion

Expressions (2), (3), (5), (9), (10) and (11) are used to investigate the impact of different electrolyte solutions and voltage stimulus on the physicochemical properties of G-actins and electrical signal propagation along F-actins.

We consider two electrolyte solutions relevant in biophysics, one representing an intracellular biological environment in physiological solution conditions (140 mM K^+^, 4 mM Cl^–^, 75 mM HPO_4_^2–^, and 012 mM Na^+^ at 310 K),^44^ whereas the other represents *in vitro* conditions^5^ (0.1 M K^+^ and 0.1 M Cl^–^ at 298 K). Additionally, we consider both 0.05 V and 0.15 V peak voltage inputs in order to simulate the typical electric potential used in cells and single microfilament experiments. Further details on the approach are provided in the ESI† document.

### 4.1. Model's parameters

Under the conditions considered in this article, the Debye length reveals a wider electrical double layer formation in the intracellular condition than *in vitro* condition, namely
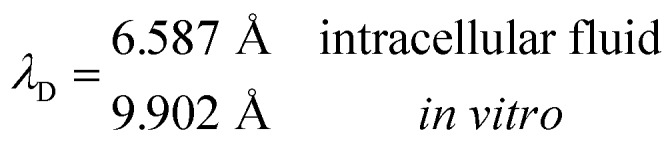
whereas, a value of the same order *𝓁*_b_ = 6.738 Å is obtained in both electrolytes for the Bjerrum length. We note that this parameter determines the length scale below, and which electrostatic correlations are important. Additionally, our approach predicts the following effective conductivity relative to the conventional bulk value approximation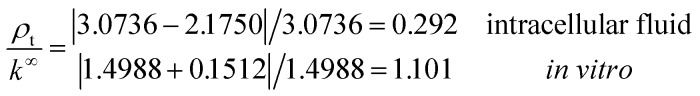 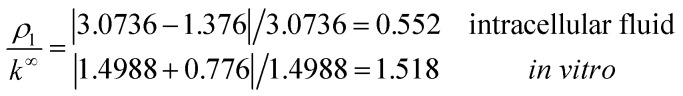
with corresponding resistances
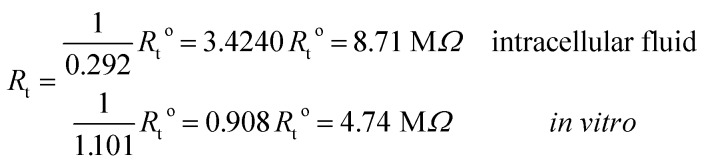
 
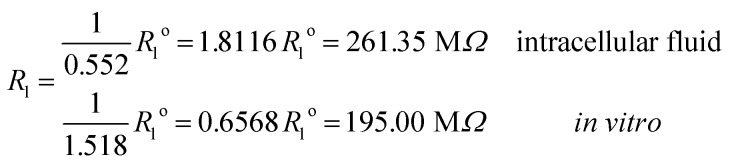
and impedances
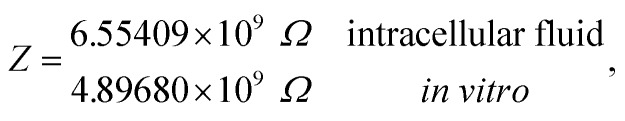


These results on ionic conductivity indicate an increase (decrease) of the resistances in typical intracellular (*in vitro*) environments compared to the corresponding bulk values. Such behavior, which is not captured by previous work,^24^ arises from a non-trivial balance (competition) between migration and convection forces, as well as, the monomeric EDL properties. These corrections to the conventional bulk approximation depend on the electrical double layer thickness, Debye length, electrochemistry (particles electrophoresis mobility, valence, bulk density and size, solvent viscosity and dielectric permittivity) and the monomer surface charge density and size.

**Fig. 4:**
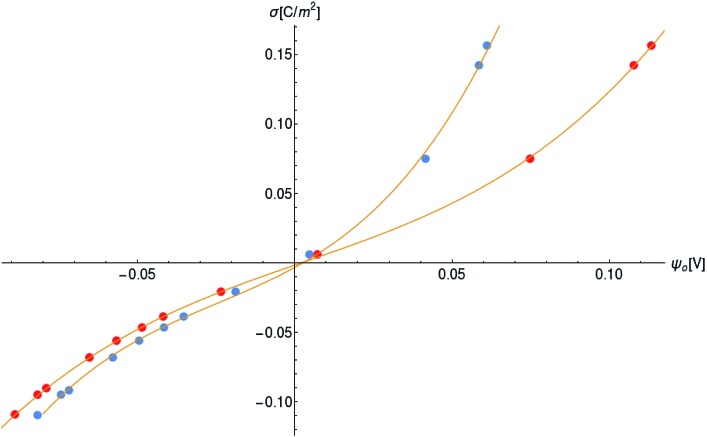
Monomer surface charge density as a function of the surface electric potential. Blue and red circles represent the data for intracellular and *in vitro* conditions, respectively.

Another neoteric result of this work is the prediction for the non-linear charge accumulation due to the linear monomeric capacitance behavior, namely *C*(*V*) = *C*_0_(1 – *bV*). To obtain the numerical values of the parameters *C*_0_ and *b*, we correlate the set of surface charge densities values *σ* predicted by the Cong model with the set of surface electrical potentials values *ψ*_o_ predicted by CSDFT. We use the Fit function provided by mathematica software^45^ to generate a cubic fitting polynomial between these two parameters as shown in Fig. 4. These curves, when used to calculate the slope analytically, generate the following value for *C*_0_ and *b*
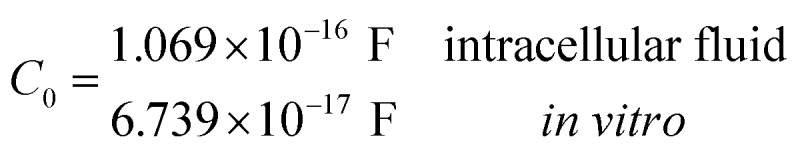
 
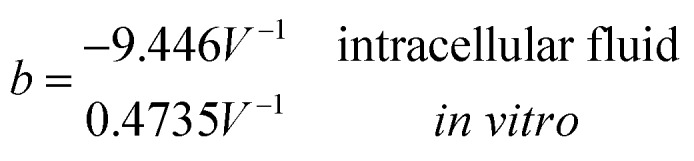


Our results on the capacitor show a remarkable increase in the linear capacitance for the intracellular condition, which has a high impact on the monomer's ability to accumulate electric energy in the capacitor. Additionally, the parameter *b* is negative for the intracellular condition, whereas it is positive for the *in vitro* condition. Accordingly, the non-linearity of the charge accumulated in the capacitor mimics the behavior of a nMOS varactor in accumulation mode and a diode^46^ in our electric circuit unit model for the intracellular and *in vitro* conditions, respectively. We note that the sign of the parameter *b* also affects the polarization of the transmission line voltage (soliton) 
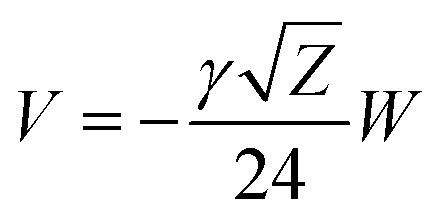
. Certainly, the following calculations16
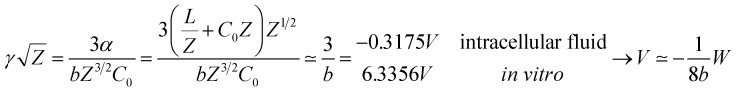
reveal electrical impulses propagating upright and down for *in vitro* and intracellular conditions, respectively. The subsequent substitution of the values obtained for *b* into eqn (10) yields *Ω*_0_ = 1.3745 and *Ω*_0_ = 2.3810 (intracellular condition) and *Ω*_0_ = 0.30772 and *Ω*_0_ = 0.5322 (*in vitro* condition), for 0.05 V and 0.15 V voltage inputs, respectively. These results clearly demonstrate the high impact of the non-linear parameter *b* in the linear relationship between the unperturbated soliton amplitude and the voltage input.

### 4.2. Electrical signal propagation

We use expressions (9)–(11) along with the numerical values for the parameters obtained previously to characterize the kern propagation velocity, shape and attenuation of the electrical impulse along an actin filament under a variety of conditions.

**Fig. 5:**
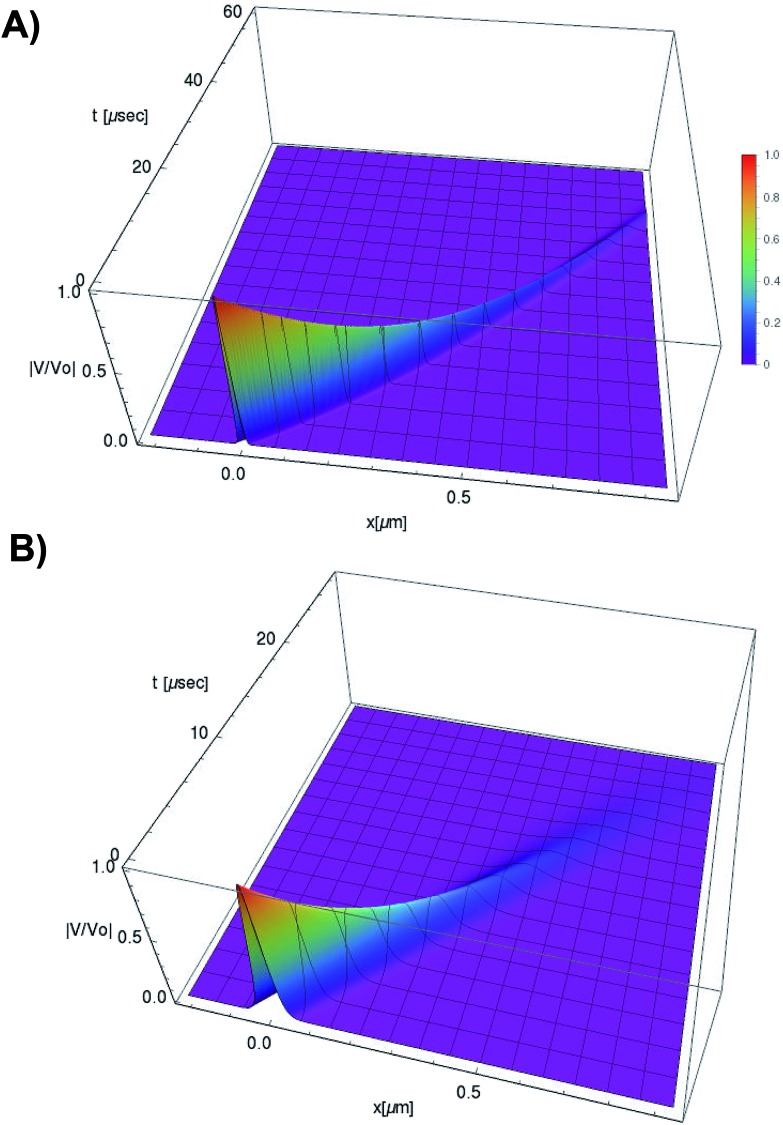
Normalized soliton solution 
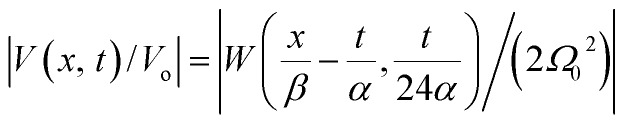
 for 0.15 V input voltage peak. (a) Soliton traveling along F-actin in intracellular conditions, (b) Soliton traveling along F-actin in in-vitro conditions.

In Fig. 5, we illustrate the propagation of normalized electrical signals with a 0.15 V voltage peak input for both intracellular and *in vitro* conditions. Our results show a similar soliton range in the order of a micron for both conditions. On the other hand, the soliton vanishing time is around 2 times longer in the intracellular condition. This result clearly shows a high impact of the biological environment on the average kern propagation velocity and attenuation.

**Fig. 6:**
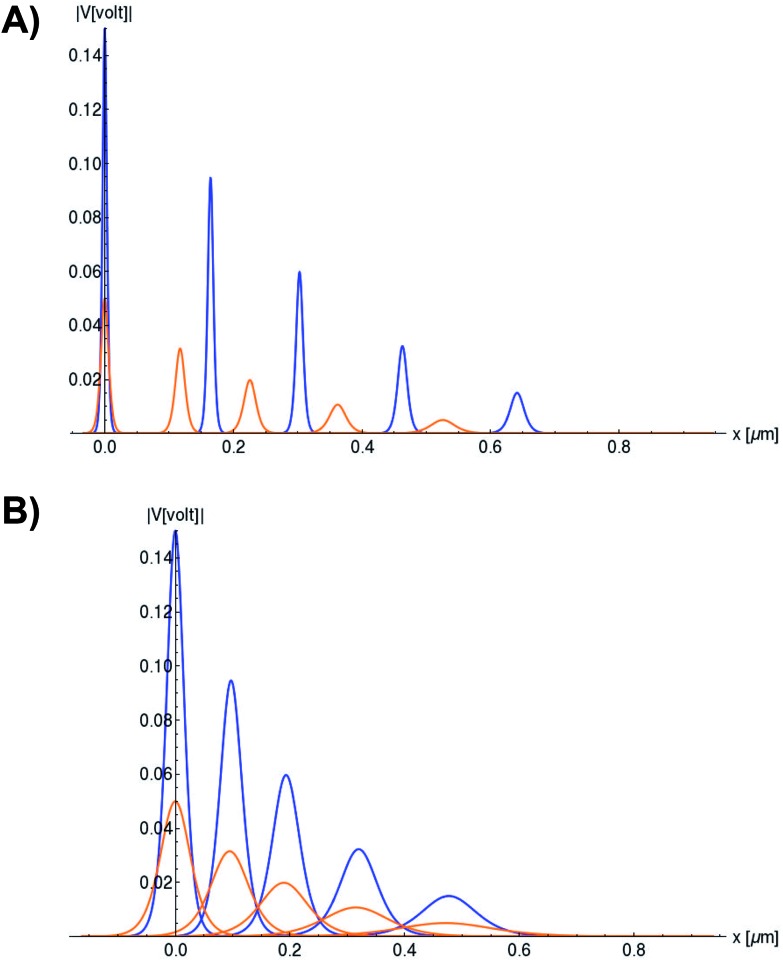
Snapshots of the normalized soliton solution 
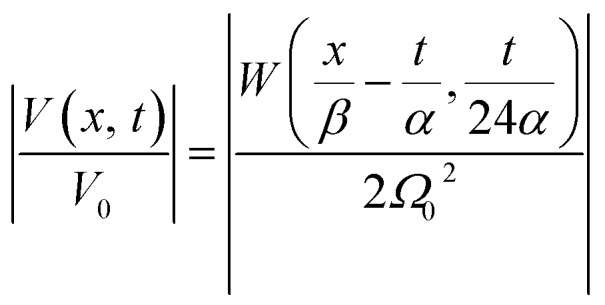
. Orange and blue colors represent the electrical signal impulse generated by 0.05 V and 0.15 V input voltage peaks, respectively. (a) Soliton profile along F-actin in intracellular conditions. The first, second, third, fourth and fiftieth peaks (same color) appearing from left to right correspond to the following snapshots *t* = 0s,*t* = 6s, *t* = 12s, *t* = 30s, and *t* = 60s, respectively. (b) Soliton profile along F-actin in *in vitro* conditions. The first,second, third, fourth and fiftieth peaks (same color) appearing from left to right correspond to the following snapshots *t* = 0s,*t* = 2:8s, *t* = 5:7s, *t* = 14:3s, and *t* = 28:6s, respectively.

**Fig. 7:**
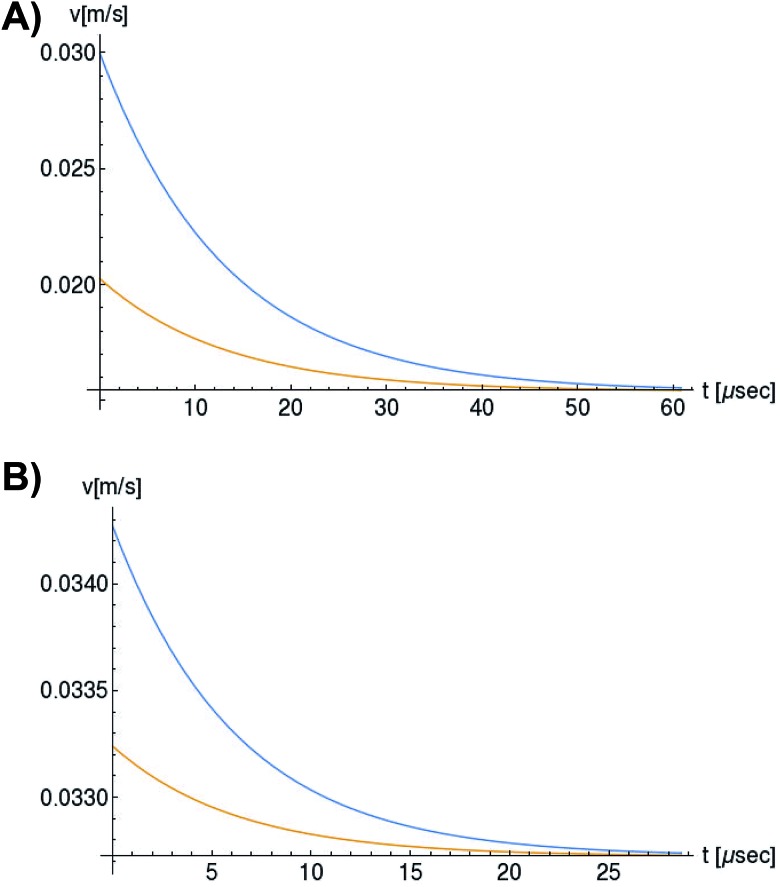
Orange and blue colors represent the propagation velocity of the electrical signal impulse generated by 0.05 V and 0.15 V input voltage peaks, respectively. (a) Soliton propagation velocity in intracellular conditions, (b) Soliton propagation velocity in *in vitro* conditions.

**Fig. 8:**
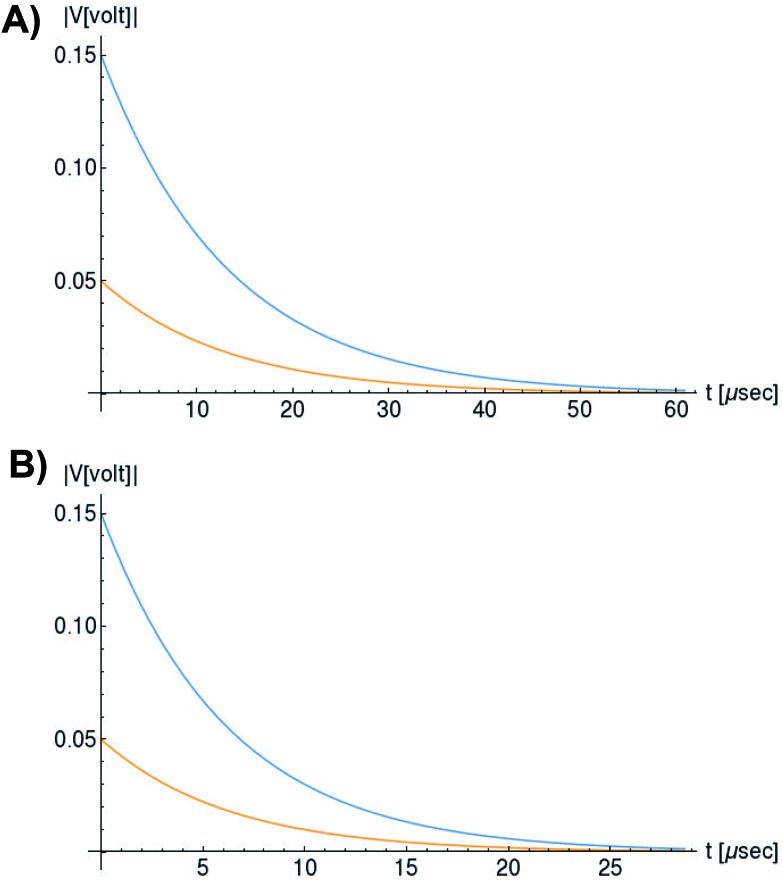
Orange and blue colors represent the electrical signal impulse amplitude generated by 0.05 V and 0.15 V input voltage peaks, respectively. (a) Soliton peak attenuation in intracellular conditions, (b) Soliton peak attenuation in *in vitro* conditions.

In Fig. 6, we compare several equitemporal snapshots of the soliton profile along F-actin for both electrolyte conditions and voltage peak inputs. In both biological conditions, the electrical impulse shape is wider for lower voltage inputs. Although, when comparing to each other, the shape of the soliton is narrower for the intracellular condition than for the *in vitro* condition. The shift between consecutive blue and orange peak positions shown in Fig. 6 indicates that solitons at higher voltage input travel faster. This is in agreement with the results displayed in Fig. 7 for the soliton kern velocity. Clearly, the filament is able to sustain the soliton propagation at almost constant kern velocity for the *in vitro* condition (see Fig. 7b), namely *v*(0) ≃ *v*_av_ = 0.0328 m s^–1^ and *v*(0) ≃ *v*_av_ = 0.0331m s^–1^ for 0.05 V and 0.15 V voltage inputs, respectively. Nevertheless, a different scenario is manifested in (Fig. 7a) where the initial kern propagation velocity for the intracellular condition is around *v*(0) = 0.03 m s^–1^ and *v*(0) = 0.02 m s^–1^ for 0.05 V and 0.15 V peak voltage inputs, respectively. The corresponding time averaged kern velocity is much lower, namely *v*_av_ = 0.01639 m s^–1^ and *v*_av_ = 0.011853 m s^–1^. This indicates a remarkable soliton propagation deceleration caused by a larger linear capacitance and non-linear parameter values, higher longitudinal ionic flow resistance, smaller electrical double layer thickness, and higher ion asymmetries (size, concentration, electrophoresis mobility, electrical valence, species number), among other factors. Additionally, our results demonstrate a higher voltage peak input that generates a higher time average kern propagation velocity. This can be understood from eqn (13) and (14), which predict an increasing propagation velocity of the electrical impulse with *Ω*_0_^2^, and consequently, with the voltage input by expression (10). On the other hand, the time average velocity comparison between both electrolyte conditions reveal that solitons from the *in vitro* condition travel, on average, faster than the intracellular condition, while the soliton peak attenuation is slower in the latter condition. This biological environment impact on electrical signal propagation is displayed in Fig. 8, which illustrates the soliton amplitude time evolution. It indicates similar vanishing time for both voltage peak inputs in each electrolyte condition. This is a consequence of the neglectable impact of the amplitude *Ω*_0_^2^ on the characteristic soliton travel time 
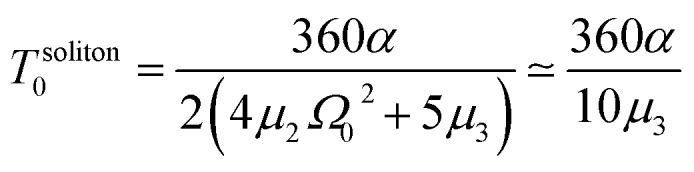
. Moreover, our results reveal a more pronounced, fast soliton attenuation decay rate at higher voltage input (blue lines in Fig. 8). This is caused by higher voltage inputs generating larger values for *Ω*_0_^2^, and larger values for the denominator in eqn (10) for *Ω*(*t*).

Overall, our results predict that the propagation of electrical signal impulses in the form of solitons are possible for a range of electrolyte solution and voltage stimulus typically present in intracellular and *in vitro* conditions. Unfortunately, we are unable to perform a quantitative comparison between our predictions and available experimental data on actin filaments,^5^ since some experimental and theoretical conditions are different. In the experimental work, it is unsure whether bundles, instead of single filaments, were used for the experiments. Further, an input square pulse was used for a duration of 800 micro seconds. Whereas, in our study, as well as, in previous work,^24^ a single filament and an instantaneous voltage stimulus are strictly considered. Although, our predictions and experimental data reveal similar trends, namely, higher voltages causes larger peaks and a faster traveling wave, which arise from a condensed ionic cloud that allows the soliton to travel along the filament.

## 5. Conclusions

In this article, we introduced an innovative multi-scale approach which accounts for the atomistic details on the protein molecular structure and biological environment, as well as their impact on electrical impulses propagating in the form of micron solitons along wild type actin filaments. The approach provides a novel, simple, accurate, approximate analytic expression for the characterization of solitons. It has been used to determine the effects of electrolyte conditions and voltage stimulus on the electrical impulse shape, attenuation and kern propagation velocity. The formulation has been shown to be capable of accounting for the details on the electrical double layer thickness and layering formation (ionic and water density distributions), the electrokinetics (particles electrophoresis mobility, valence and size, solvent viscosity and dielectric permittivity), and the monomer–electrolyte interface (surface charge density and size) on the ionic electrical conductivity and capacitance.

Our results reveal a high impact of the electrolyte condition on electrical conductivity and capacitance in G-actins. The approach predicts wider electrical double layer, lower electrical conductivity, higher linear capacitance and non-linear accumulation of charge in intracellular conditions, which play an important role on the electrical signal propagation along the actin filament. Additionally, the non-linearity of the charge accumulated in the capacitor resembles the behavior of a varactor and a diode in our electric circuit unit model for the intracellular and *in vitro* conditions, respectively. The approach also predicts different polarization of the transmission line voltage (soliton). The electrical impulse propagates upright and down for *in vitro* and intracellular conditions, respectively.

Our results also show a significant influence of the voltage input on the electrical impulse shape, attenuation and kern propagation velocity. The filament is able to sustain the soliton propagation at almost constant kern velocity for the *in vitro* condition, but it displays a remarkable deceleration for the intracellular condition, with a slower soliton peak attenuation which is more pronounced at higher voltage input. Solitons travel faster at higher voltage input, but are narrower for intracellular conditions. On the other hand, the electrical impulse shape is wider at lower voltage input and the soliton range is in the order of one micron for both electrolyte conditions. Although, the vanishing time is around 2 times longer for the intracellular condition.

Overall, our results predict that the propagation of electrical signal impulses in the form of solitons are possible for a range of electrolyte solutions and voltage stimulus typically present in intracellular and *in vitro* conditions. Our predictions are an improvement on less recent theories and in qualitative agreement with experimentally obtained data for actin filaments. One of the most important outcomes of this approach lies in the ability to determine the impact of molecular structure conformation (mutations) and physicochemical solution changes (protonations/deprotonations alterations) often present in pathological conditions in cytoskeleton filaments. This multi-scale theory may also be applicable to other highly charged rod-like polyelectrolytes with relevance in biomedicine and biophysics.^3^ Currently we are working along this direction with the ultimate goal of providing a molecular understanding for how and why age and inheritance conditions induce dysfunction and malformation in cytoskeleton filaments associated with a variety of diseases.^1^

Our predictions are an improvement on less recent theories and in qualitative agreement with experimentally obtained data for filaments.

## Conflicts of interest

There are no conflicts to declare.

## Supplementary Material

Supplementary information

## References

[cit1] dos Remedios C. and Chhabra D., Actin-Binding Proteins and Disease, Protein Reviews, Springer, New York, 2008.

[cit2] Woolf N. J. and Priel A., Nanoneuroscience: Structural and Functional Roles of the Neuronal Cytoskeleton in Health and Disease, Biological and Medical Physics, Biomedical Engineering, Springer, Berlin Heidelberg, 2009.

[cit3] Janmey P. A., Slochower D. R., Wang Yu-H., Wen Q., Cebers A. (2014). Soft Matter.

[cit4] Cantiello H. F., Patenaude C., Zaner K. (1991). Biophys. J..

[cit5] Lin E. C., Cantiello H. F. (1993). Biophys. J..

[cit6] Goldman J. E. (1983). J. Neurosci..

[cit7] Lange K. (2000). J. Theor. Biol..

[cit8] Gartzke J., Lange K. (2002). Am. J. Physiol.: Cell Physiol..

[cit9] Sundberg M., Bunk R., Albet-Torres N., Kvennefors A., Persson F., Montelius L., Nicholls I. A., Ghatnekar-Nilsson S., Omling P., Tågerud S. (2006). Langmuir.

[cit10] Arsenault M. E., Zhao H., Purohit P. K., Goldman Y. E., Bau H. H. (2007). Biophys. J..

[cit11] Galland R. Leduc P. Guérin C. Peyrade D. Blanchoin L. Théry M., Nat. Mater., 2013, 125, 416–421, , A press release CEA, CNRS, UJF, INRA has been published for this publication (February 11th, 2013): http://www2.cnrs.fr/presse/communique/2987.htm. 23396247 10.1038/nmat3569

[cit12] Patolsky F. Weizmann Y. Willner I., Nat. Mater., 2004, 310, 692–695, , Copyright – Copyright Nature Publishing Group Oct 2004; Last updated – 2014-04-30. 15359342 10.1038/nmat1205

[cit13] Korten T., Månsson A., Diez S. (2010). Curr. Opin. Biotechnol..

[cit14] Oosawa F. (1970). Biopolymers.

[cit15] Manning G. S. (1978). Q. Rev. Biophys..

[cit16] Manning G. S. (1969). J. Chem. Phys..

[cit17] Zimm B. H.. Use of the Poisson–Boltzmann Equation To Predict Ion Condensation Around Polyelectrolytes, 1986, ch. 17, pp. 212–215.

[cit18] Kolosick J. A., Landt D. L., Hsuan H. C. S., Lonngren K. E. (1974). Proc. IEEE.

[cit19] Noguchi A. (1974). Electronics and Communications in Japan.

[cit20] Lonngren K. E., Observations of solitons on non-linear dispersive transmission lines, in Solutions in Action, ed. Karl Lonngren and Alwyn Scott, Academic Press, 1978, pp. 127–152.

[cit21] Novikov S., Manakov S. V., Pitaevskii L. P., and Zakharov V. E., Theory of Solitons: The Inverse Scattering Method, Plenum, New York, 1984.

[cit22] Le Bret M., Zimm B. H. (1984). Biopolymers.

[cit23] Newman J., Electrochemical Systems, N.J. Prentice-Hall, Englewood Cliffs, ch. 1, 1973.

[cit24] Tuszyński J. A., Portet S., Dixon J. M., Luxford C., Cantiello H. F. (2004). Biophys. J..

[cit25] Satarić M. V., Ilić D. I., Ralević N., Tuszynski J. A. (2009). Eur. Biophys. J..

[cit26] Sekulić D. L., Satarić B. M., Tuszyński J. A., Satarić M. V. (2011). Eur. Phys. J. E: Soft Matter Biol. Phys..

[cit27] Sekulić D. L., Satarić B. M. (2015). Facta Universitatis, Series: Electronics and Energetics.

[cit28] Kornyshev A. A. (2007). J. Phys. Chem. B.

[cit29] en Jiang D., Meng D., Wu J. (2011). Chem. Phys. Lett..

[cit30] Lamperski S., Pluciennik M., Outhwaite C. W. (2015). Phys. Chem. Chem. Phys..

[cit31] Warshavsky V., Marucho M. (2016). Phys. Rev. E: Stat., Non-linear, Soft Matter Phys..

[cit32] Cong Y., Topf M., Sali A., Matsudaira P., Dougherty M., Chiu W., Schmid M. F. (2008). J. Mol. Biol..

[cit33] Newman J., Electrochemical Systems, N. J. Prentice-Hall, Englewood Cliffs, ch. 9, 1973.

[cit34] Ovanesyan Z., Medasani B., Fenley M. O., Guerrero-García G. I., de la Cruz M. O., Marucho M. (2014). J. Chem. Phys..

[cit35] Medasani B., Ovanesyan Z., Thomas D. G., Sushko M. L., Marucho M. (2014). J. Chem. Phys..

[cit36] Hunley C., Marucho M. (2017). Phys. Chem. Chem. Phys..

[cit37] Ovanesyan Z., Aljzmi A., Almusaynid M., Khan A., Valderrama E., Nash K. L., Marucho M. (2016). J. Colloid Interface Sci..

[cit38] Dolinsky T. J., Nielsen J. E., Andrew McCammon J., Baker N. A. (2004). Nucleic Acids Res..

[cit39] Voss N. R., Gerstein M. (2010). Nucleic Acids Res..

[cit40] Sekulić D. L. and Zivanov M. B., Computational study on soliton-like pulses in the non-linear rlc transmission lines, in 2012 Proceedings of the 35th International Convention2012 Proceedings of the 35th International Convention MIPROMIPRO, 2012, pp. 228–232.

[cit41] Ablowitz M., Segur H., Solitons and the Inverse Scattering Transform, SIAM, Philadelphia,1981.

[cit42] Mohamad Jawada A. J. (2014). Sci. Iran., Trans. D.

[cit43] Karpman V. I., Maslov E. M. (1977). Zh. Eksp. Teor. Fiz..

[cit44] Lodish H., Berk A., Zipursky S. L., Lodish H., Matsudaira P., Baltimore D., and Darnell J., Intracellular Ion Environment and Membrane Electric Potential, Molecular Cell Biology, W. H. Freeman, New York, 4th edn, 2000, ch. 15.4.

[cit45] Wolfram Research, Inc., Mathematica Version 11.0, 2016.

[cit46] Afshari E., Hajimiri A. (2005). IEEE J. Solid-State Circuits.

