# Supplementary material for: A multi-scale approach to describe electrical impulses propagating along actin filaments in both intracellular and in vitro conditions
Source: RSC Adv. 2018 Mar 28;8(22):12017–28. doi: 10.1039/c7ra12799e (PMC6369918; doi:10.1039/c7ra12799e)
Supplement: RA-008-C7RA12799E-s001 [file RA-008-C7RA12799E-s001.pdf]

Electronic Supplementary Material (ESI) for RSC Advances.
This journal is © The Royal Society of Chemistry 2018

Appendix A. Mathematical derivation for the master equations introduced in the article

Longitudinal and Transversal Ionic Flow Resistances

The transport law and assumptions introduced in section 2.2 provide the following equation for the axial electric surface current density[1]

$$i_z(r) = k(r)E_z + v_z(r)\rho_e(r) \qquad r \geq r_\xi > R \tag{A.1}$$

This expression depends on the electrolyte conductivity

$$k(r) = F^2 \sum_i z_i^2 u_i c_i(r) \tag{A.2}$$

and the total charge density distribution

$$\rho_e(r) = F \sum_i z_i c_i(r) \tag{A.3}$$

In the previous expressions, F is Faraday's constant, whereas, the mobility, valence, and concentration of ion species i are represented by u_i, z_i, and $c_i(r)$, respectively.

Moreover, we combine Poisson and the Navier-Stokes equations to obtain the following expression for the axial velocity profile

$$v_z(r) = \frac{\epsilon E_z}{\mu}\left[\phi(r) - \phi(r_\xi)\right] \qquad r \geq r_\xi. \tag{A.4}$$

This expression indicates that under the action of a uniform axial electric field, the velocity profile of the fluid is proportional to the radial electric potential drop $\phi(r) - \phi(r_\xi)$. In the latter equation, $\epsilon = 7.0832 \cdot 10^{-10}\frac{F}{m}$ and $\mu = 0.00089\frac{Kg}{m.s}$ represent the absolute bulk permittivity and viscosity parameters, respectively. On the other hand, the radial electric potential $\phi(r)$ generated by the total charge density distribution is dictated by the Poisson equation

$$\rho_e(r) = -\frac{\epsilon}{r}\frac{\partial}{\partial r}\left(r\frac{\partial \phi(r)}{\partial r}\right) \tag{A.5}$$

To obtain an analytic solution for the electric potential we use a Boltzmann distribution and Debye-Hückel (linearized PB) approximation for the ion density distributions

$$c_i(r) = c_i^\infty \exp\left[-\frac{z_i F\phi(r)}{RT}\right] \approx c_i^\infty\left(1 - \frac{z_i F\phi(r)}{RT}\right), \qquad \left|\frac{z_i F\phi(r)}{RT}\right| \ll 1 \tag{A.6}$$

where c_i^∞ is the bulk concentration of species i $\left[\frac{mol}{m^3}\right]$, R the gas constant, and T the electrolyte temperature. After substitution of eqn (A.6) into eqn (A.3), the use of the bulk electroneutrality condition ($\sum_i z_i c_i^\infty = 0$), and the replacement of the resulting expression into eqn (A.5), we obtain the expression for $\phi(r)$ given by eq.(1) in the article, namely

$$\phi(r) = \frac{\sigma\lambda_D}{\epsilon}\frac{K_0\left(\frac{r}{\lambda_D}\right)}{K_1\left(\frac{r_\xi}{\lambda_D}\right)} \qquad r > R \tag{A.7}$$

where K is the modified Bessel function of the second kind, λ_D represents the Debye length $\lambda_D = \left(\epsilon RT/\left(F^2 \sum_i z_i^2 c_i^\infty\right)\right)^{\frac{1}{2}}$, σ the monomer surface charge density, and r_ξ is the radius predicted by the cylindrical model.

We numerically evaluate the mean electric potential obtained for both electrolytes using the non-linearized and linearized PB solutions for validation purposes (see figure A.1). We obtained a maximum deviation of 9 %, which is within the range of values accepted for this kind of approximation.

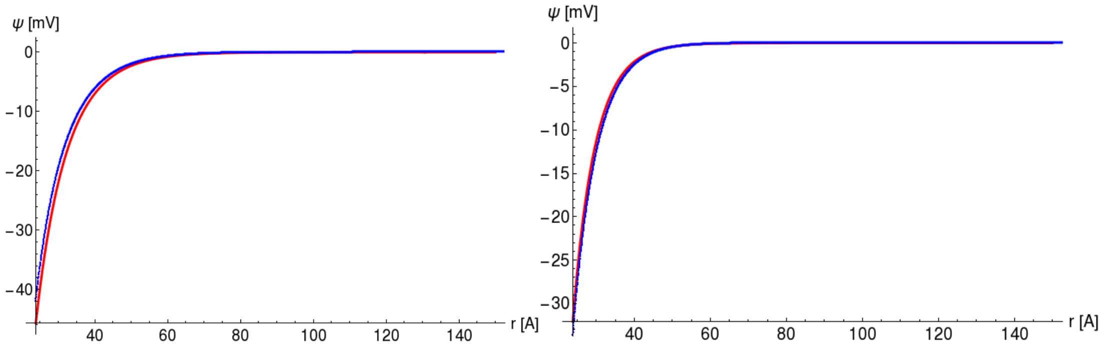

(a) Intracellular conditions.

Figure A.1: Mean electrostatic potential as a function of the separation distance. Comparison between numerical NLPB (blue color) and analytic LPB (red color) solutions. The figures to the left and right sides correspond to the in-vitro and intracellular electrolyte solutions, respectively.

The approximate analytic solution obtained for the electric potential $\phi(r)$ is subsequently replaced into eqn (A.6), (A.5), (A.4), and (A.2) to get an analytic solution for the surface current density. This solution is integrated over the Bjerrum length $\ell_b = \frac{e^2}{4\pi\varepsilon\varepsilon_0 k_b T} = 6.738\mathring{A}$. In the latter expressions, $\varepsilon = 80$ is the relative bulk solvent dielectric constant; $\varepsilon_0 = 8.854 \cdot 10^{-12} \left[\frac{F}{m}\right]$ the vacuum permittivity; $k_b = 1.381 \cdot 10^{-23} \left[\frac{J}{K}\right]$ the Boltzmann's constant, and T the temperature in Kelvin degree. The integration yields the following expression for the total longitudinal ionic current

$$\frac{I_l}{2\pi} = E_z \int_{r_\xi}^{\ell_b + r_\xi} r k(r) dr + \int_{r_\xi}^{\ell_b + r_\xi} r v(z) \rho_e(r) dr \quad \text{(A.8)}$$

After performing some algebra and integral calculations using mathematica11.1 software[2] expression A.8 becomes

$$I_l = E_z \pi \left((\ell_b + r_\xi)^2 - r_\xi^2\right) \{k^\infty + \triangle k_l\} = \frac{|\Delta V|}{\ell} \pi \left((\ell_b + r_\xi)^2 - r_\xi^2\right) \{k^\infty + \triangle k_l\} \quad \text{(A.9)}$$

where k^∞ is the bulk electrolyte conductivity

$$k^\infty = F^2 \sum_i z_i^2 u_i c_i^\infty, \quad \text{(A.10)}$$

$\triangle k_l$ the correction predicted by our approach

$$\triangle k_l = -\frac{2F^3\sigma\lambda_D^2 r_\xi \sum_i z_i^3 u_i c_i^\infty}{\epsilon RT((\ell_b+r_\xi)^2 - r_\xi^2)}\left(1 - \frac{(\ell_b+r_\xi)\, K_1\left(\frac{\ell_b+r_\xi}{\lambda_D}\right)}{r_\xi K_1\left(\frac{r_\xi}{\lambda_D}\right)}\right) + \tag{A.11}$$

$$\frac{r_\xi^2\sigma^2}{\mu\left((\ell_b+r_\xi)^2 - r_\xi^2\right)} G\left(\ell_b, r_\xi, \lambda_D\right)$$

and G is the following analytic function

$$G\left(\ell_b, r_\xi, \lambda_D\right)\left[K_1\left(\frac{r_\xi}{\lambda_D}\right)\right]^2 = \left\{\left(K_0\left(\frac{r_\xi}{\lambda_D}\right)^2 - K_1\left(\frac{r_\xi}{\lambda_D}\right)^2\right) + 2\frac{\lambda_D}{r_\xi}K_0\left(\frac{r_\xi}{\lambda_D}\right)K_1\left(\frac{r_\xi}{\lambda_D}\right)\right.$$

$$\left. - \frac{(\ell_b+r_\xi)^2\left(K_0\left(\frac{(\ell_b+r_\xi)}{\lambda_D}\right)^2 - K_1\left(\frac{(\ell_b+r_\xi)}{\lambda_D}\right)^2\right) - 2\frac{\lambda_D(\ell_b+r_\xi)}{r_\xi^2}K_0\left(\frac{(\ell_b+r_\xi)}{\lambda_D}\right)K_1\left(\frac{(\ell_b+r_\xi)}{\lambda_D}\right)}{r_\xi^2}\right\}.$$

Finally, we use Ohm's law, which relates the axial voltage drop ΔV and the total electric current I as $R_l = |\Delta V/I_l|$, to obtain the expression for the longitudinal ionic flow resistance R_l introduced in the article by eq.(2), namely

$$R_l = \frac{\ell}{\pi\left((\ell_b+r_\xi)^2 - r_\xi^2\right)|k^\infty + \triangle k_l|} = \frac{\ell}{S_l \varrho_l}, \tag{A.12}$$

A similar approach is used for the transversal ionic flow resistance calculations. The transport's law and the assumptions introduced in section 2.2 provide the following equation for the radial surface current density

$$i_r(r) = -F\sum_i z_i\left(F z_i u_i c_i(r)\frac{\partial\phi(r)}{\partial r}\right) \tag{A.13}$$

Since this expression does not depend on the axial and azimuthal coordinates, the total radial current I_r passing from the inner to the outer layer is obtained by multiplying the radial surface current density by the lateral monomeric surface layer area at a section r in the solution, e.g. $I_r = i_r(r)2\pi\ell r$.[1] Since I_r is a constant independent of position, this expression can be integrated across the electrical double layer to obtain

$$\int_{r_\xi}^{\ell_b+r_\xi}\frac{I_r}{2\pi\ell r}dr = \int_{r_\xi}^{\ell_b+r_\xi} i_r(r)dr = -k^\infty\int_{r_\xi}^{\ell_b+r_\xi}\frac{\partial\phi(r)}{\partial r}dr + \frac{F^3}{RT}\sum_i z_i^3 u_i c_i^\infty\int_{r_\xi}^{\ell_b+r_\xi}\phi(r)\frac{\partial\phi(r)}{\partial r}dr \tag{A.14}$$

Therefore,

$$\frac{I_r \ln\left(\frac{\ell_b+r_\xi}{r_\xi}\right)}{2\pi\ell} = -k^\infty\left[\phi(\ell_b+r_\xi)-\phi(r_\xi)\right] + \frac{F^3}{2RT}\sum_i z_i^3 u_i c_i^\infty\left[\phi^2(\ell_b+r_\xi)-\phi^2(r_\xi)\right]$$

After some algebra we obtain a linear dependence between the total radial current I_r and the electric potential drop across the electrical double layer $\Delta\phi \equiv \phi(r_\xi)-\phi(\ell_b+r_\xi)$ following the Ohm-like law equation

$$\frac{I_r \ln\left(\frac{\ell_b+r_\xi}{r_\xi}\right)}{2\pi\ell\left[k^\infty - \frac{F^3}{2RT}\sum_i z_i^3 u_i c_i^\infty\left[\phi(\ell_b+r_\xi)+\phi(r_\xi)\right]\right]} \equiv I_r R_t = \Delta\phi$$

where

$$R_t = \frac{\ln\left(\frac{\ell_b+r_\xi}{r_\xi}\right)}{2\pi\ell\left|k^\infty - \frac{F^3}{2RT}\sum_i z_i^3 u_i c_i^\infty\left[\phi(\ell_b+r_\xi)+\phi(r_\xi)\right]\right|} = \frac{\ell_b}{2\pi\ell r_\xi\left|k^\infty+\triangle k_t\right|} = \frac{\ell_b}{S_t\varrho_t}, \qquad \text{(A.15)}$$

is the expression for radial ionic flow resistance introduced in the article by eq. (3). In the later expression $\triangle k_t$ represents the correction to the bulk electrolyte conductivity

$$\triangle k_t = k^\infty\left\{\frac{\ell_b}{r_\xi \ln\left(\frac{\ell_b+r_\xi}{r_\xi}\right)} - 1\right\} - \frac{\ell_b\sigma\lambda_D F^3\sum_i z_i^3 u_i c_i^\ell}{2r_\xi RT\epsilon K_1\left(\frac{r_\xi}{\lambda_D}\right)\ln\left(\frac{\ell_b+r_\xi}{r_\xi}\right)}\left[K_0\left(\frac{(\ell_b+r_\xi)}{\lambda_D}\right)+K_0\left(\frac{r_\xi}{\lambda_D}\right)\right] \qquad \text{(A.16)}$$

Capacitance

CSDFT characterizes the polyelectrolyte properties of the an actin filament by the effective molecular radius R and uniform bare surface charge density σ, obtained in this work from the Cong molecular structure model, whereas the biological environment is represented by an electrolyte (either alkaline, acid or neutral) solution comprised of ionic species (characterized by crystal radius,[3] charge and bulk concentration), explicit water molecules (characterized by neutral ions at experimental size (2.75Å) and bulk concentration ($55.56M$)). We assume that the filament, monomer radius and surface charge density are approximately similar. We consider a monomer exposed to different pH levels (alkaline, acid and neutral levels) in the solution. For each pH level, we use the Cong molecular structure model and perform titration calculations to predict the corresponding surface charge density value σ, as explained previously. Subsequently, we apply CSDFT to predict the surface electrical potential ψ_o for each of the values σ obtained in the previous step. These two sets of parameter values $[\sigma, \psi_o]$, when correlated using a cubic fitting polynomial curve, determine the slope analytically from where the following expression for the differential capacitance C_d is obtained

$$C_d = \frac{d\sigma}{d\psi_o} = \widehat{C}_o\left(1 - 2\widehat{b}\psi_o + 3\widehat{c}\psi_o^2 + \mathcal{O}\left(\psi_o^3\right)\right) \qquad \text{(A.17)}$$

Integration of this expression with respect to the electric potential along the voltage drop V across the electrical double layer leads to the expression for the total charge accumulated in the capacitor introduced in the article by eq.(4), namely[4]

$$Q = 2\pi r_\xi \ell \widehat{C}_o \left(V - \widehat{b}V^2\right) = C_o\left(V - bV^2\right) = VC_o\left(1 - bV\right) = VC\left(V\right) \tag{A.18}$$

Electrical Signal Propagation

The current conservation law at the node A between the cells "$m-1$" and "m", provides the following equation (see Fig. 3 in the article)

$$I_{m-1} - I_m = \frac{\partial Q_m}{\partial t} \tag{A.19}$$

where $\partial Q_m/\partial t$ represents the current across the capacitor in the cell "m". By replacing eqn (4) into eqn (A.19) we have

$$I_{m-1} - I_m = C_o\left(\frac{\partial V_m}{\partial t} - 2bV_m\frac{\partial V_m}{\partial t}\right) \tag{A.20}$$

On the other hand, Kirchhoff's voltage law along the circuit ABCD generates the following expression (see Fig. 3 in the article)

$$v_m - v_{m+1} = L\frac{\partial I_m}{\partial t} + I_m R_l \tag{A.21}$$

where v_m is given by

$$v_m = R_t\left(I_{m-1} - I_m\right) + V_o + V_m \tag{A.22}$$

and V_o represents a constant DC bias electric potential. Further substitution of eqn (A.22) into eqn (A.21) yields

$$L\frac{\partial I_m}{\partial t} + I_m R_l = R_t\left(I_{m-1} - 2I_m + I_{m+1}\right) + \left(V_m - V_{m+1}\right) \tag{A.23}$$

The later equation can be written in terms of the characteristic impedance of the electrical circuit unit Z and the new function $U_m(t)$, where as usual:[5] $Z^{-1/2}U_m = I_m$ and $Z^{1/2}U_m = V_m$. In this article, the characteristic impedance is estimated as follows

$$Z \simeq \sqrt{R_{equiv}^2 + X_{equiv}^2} \tag{A.24}$$

where $R_{equiv} = R_l + R_t$, $X_{equiv} = \frac{T_o^{G-actin}}{2\pi C_o}$, and $T_o^{G-actin}$ is a parameter characterizing the electrical circuit unit time scale.

These expressions, when replaced into eqn (A.20) and (A.23), lead to the coupled equations (6) and (7) introduced in the article, namely

$$U_{m-1} - U_m = C_0 Z\left(\frac{\partial U_m}{\partial t} - 2bZ^{1/2}U_m\frac{\partial U_m}{\partial t}\right) \tag{A.25}$$

$$U_m - U_{m+1} = Z^{-1}\left[L\frac{\partial U_m}{\partial t} + U_m R_l - R_t\left(U_{m+1} - 2U_m + U_{m-1}\right)\right] \tag{A.26}$$

Using the continuum approximation $U_m(t) \backsimeq U(x,t)$ with a Taylor series in terms of the parameter ℓ mentioned in the article, we obtain the following expansion for $U_{m\pm1}(t)$

$$U_{m\pm1} = U(x\pm\ell,t) \backsimeq U \pm \ell\frac{\partial U}{\partial x} + \frac{\ell^2}{2}\frac{\partial^2 U}{\partial x^2} \pm \frac{\ell^3}{3!}\frac{\partial^3 U}{\partial x^3} \tag{A.27}$$

Consequently,

$$U_{m+1} - 2U_m + U_{m-1} = \ell^2\frac{\partial^2 U}{\partial x^2} \tag{A.28}$$

$$U_{m-1} - U_{m+1} = -2\ell\frac{\partial U}{\partial x} - \frac{\ell^3}{3}\frac{\partial^3 U}{\partial x^3} \tag{A.29}$$

By summing eqn (A.25), (A.26), and using eqn (A.28) and (A.29) we have

$$Z^{-1}\left[L\frac{\partial U}{\partial t} + UR_l - R_t\left(\ell^2\frac{\partial^2 U}{\partial x^2}\right)\right] + C_0\left(Z\frac{\partial U}{\partial t} - 2bZ^{3/2}U\frac{\partial U}{\partial t}\right) = -2\ell\frac{\partial U}{\partial x} - \frac{\ell^3}{3}\frac{\partial^3 U}{\partial x^3} \tag{A.30}$$

The later equation can be conveniently rewritten as follows

$$\left[\frac{L}{Z} + C_0 Z\right]\frac{\partial U}{\partial t} + \frac{R_l}{Z}U - \frac{R_t\ell^2}{Z}\frac{\partial^2 U}{\partial x^2} - 2bZ^{3/2}C_0 U\frac{\partial U}{\partial t} + 2\ell\frac{\partial U}{\partial x} + \frac{\ell^3}{3}\frac{\partial^3 U}{\partial x^3} = 0 \tag{A.31}$$

The master equation (A.31) can be solved for $U(x,t) = U(x(\xi,\tau),t(\tau)) = U(\xi,\tau)$ in terms of dimensionless variables

$$\xi = \frac{x}{\beta} - \frac{t}{\alpha}, \qquad \tau = \frac{t}{24\alpha}, \qquad \text{where} \qquad \alpha = \frac{L}{Z} + C_0 Z > 0, \quad \text{and} \quad \beta = 2\ell, \tag{A.32}$$

After some manipulations and using the following relationships $\frac{\partial}{\partial t} = \frac{1}{\alpha}(\frac{\partial}{24\partial\tau} - \frac{\partial}{\partial\xi})$, $\frac{\partial}{\partial x} = \frac{1}{\beta}\frac{\partial}{\partial\xi}$, $\frac{\partial^2}{\partial x^2} = \frac{1}{\beta^2}\frac{\partial^2}{\partial\xi^2}$ and $\frac{\partial^3}{\partial x^3} = \frac{1}{\beta^3}\frac{\partial^3}{\partial\xi^3}$, we have

$$\frac{\gamma\partial U}{24\partial\tau} + \frac{\gamma R_l}{Z}U - \frac{\gamma R_t}{4Z}\left(\frac{\partial^2 U}{\partial\xi^2}\right) + 6U\left(\frac{\partial U}{\partial\xi} - \frac{\partial U}{\partial\tau}\right) + \frac{\gamma}{24}\frac{\partial^3 U}{\partial\xi^3} = 0 \tag{A.33}$$

where $\gamma = \frac{3\alpha}{bZ^{3/2}C_0}$. Since we look for slow changes on the time evolution in the electrical impulse solution of eqn (A.33), $\frac{1}{24}\frac{\partial U}{\partial\tau} \ll \frac{\partial U}{\partial\xi}$, the time rate in the electrical impulse is considered to be much slower than on the traveling variable. As a result, an analytic solution of eqn (A.33) can

be obtained by performing the change, $U = -\frac{\gamma}{24}W$ and defining new parameters $\mu_2 = \frac{6R_t}{Z}$, $\mu_3 = \frac{24R_l}{Z}$. Accordingly, eqn (A.33) becomes the well-known perturbated Korteweg-de Vries (pKdV) differential equation[6,7] introduced in the article by eq. (8), namely

$$\frac{\partial W}{\partial \tau} - 6W\frac{\partial W}{\partial \xi} + \frac{\partial^3 W}{\partial \xi^3} = \mu_2 \frac{\partial^2 W}{\partial \xi^2} - \mu_3 W \equiv P\left(W\right) \tag{A.34}$$

The left and right sides in the later equation represent the regular KdV equation[8] and the corresponding perturbation, respectively.

An initial pulse $W\left(\xi, 0\right)$ in the transmission line may decay into a sequence of solitons and a tail. In this work, we will consider single soliton solutions only. In doing so, we assume that the perturbation is so small that it has a negligible influence on the soliton formation. Therefore, the perturbation will manifest itself by affecting the soliton only after an extended amount of time from its origination. Thus, the mutual interaction between solitons becomes unimportant when the soliton movement is of the order of its length. In what follows we consider this problem in the first approximation. In this case, the solution of equation (A.34) for non-dissipative systems $(R_l = R_t = 0)$ represents a non perturbated pulse soliton of the regular KdV equation ([9])

$$W_{np}(\xi, \tau) = -2\Omega_0^2 sech^2\left[\Omega_0\left(\xi - 4\Omega_0^2\tau\right)\right] \tag{A.35}$$

with dimensionless constant voltage amplitude $2\Omega_0^2$ and propagation velocity $4\Omega_0^2$. Solitary-wave solutions that propagate without changing form may also be expected due to a balance between non-linearity and dispersion (e.g. $|P(W)| \simeq 0$). Hence, requiring the effect of the perturbing terms on the shape of the soliton cancel each other out.[10] Otherwise, when μ_2 and/or μ_3 are not zero, equation (A.35) is no longer the solution of the perturbed KdV equation.[8,9,11,12]

In this analysis, we look for an analytic solution of eqn (A.35) in the framework of the perturbation theory on the basis of the adiabatic approximation.[9] In that case, the solution is a soliton pulse $W(\xi, \tau)$ in the form introduced in the article by equation (9), namely

$$W(\xi, \tau) = -2\left[\Omega\left(\tau\right)\right]^2 sech^2\left[\Omega\left(\tau\right)\left(\xi - \eta\left(\tau\right)\right)\right] \tag{A.36}$$

where $\Omega\left(\tau\right)$ and $\eta\left(\tau\right)$ satisfy the following equations

$$\frac{d\Omega}{d\tau} = -\frac{1}{4\Omega}\int\limits_{-\infty}^{\infty} P(W) sech^2 z dz, \tag{A.37}$$

$$\frac{d\eta}{d\tau} = 4\Omega^2 - \frac{1}{4\Omega^3}\int\limits_{-\infty}^{\infty} P(W)\left[z + \frac{1}{2}sinh(2z)\right] sech^2 z dz, \tag{A.38}$$

$z = \Omega\left(\tau\right)\left(\xi - \eta\left(\tau\right)\right)$ and $P(W)$ is the perturbation term defined in eqn (A.34). The calculation of the integrals appearing in eqn (A.37) and (A.38) provide the following analytic solutions

$$\begin{aligned} \Omega\left(\tau\right) &= \Omega_0 \sqrt{\frac{\exp\left(-\frac{4\tau\mu_3}{3}\right)}{1+\frac{4\mu_2\Omega_0^2}{5\mu_3}\left(1-\exp\left(-\frac{4\tau\mu_3}{3}\right)\right)}} \equiv \Omega_0 M_1\left(\tau\right) \\ &\simeq \Omega_0\left(1 - \tfrac{2}{15}\left(4\mu_2\Omega_0^2 + 5\mu_3\right)\tau + \mathcal{O}\left(\left(\tfrac{4\tau\mu_3}{3}\right)^2\right)\right) \end{aligned} \tag{A.39}$$

$$\eta(\tau) = -\tfrac{5}{4\mu_2}\left[4\mu_3\tau + 3\log\left[5\mu_3\right]\right]$$
$$-\tfrac{5}{4\mu_2}\left[-3\log\left[-4\Omega_0^2\mu_2 + \exp\left(\tfrac{4\tau\mu_3}{3}\right)\left(4\Omega_0^2\mu_2 + 5\mu_3\right)\right]\right] \equiv 4\Omega_0^2\tau M_2\left(\tau\right)$$
$$\simeq 4\Omega_0^2\tau\left(1 - \frac{2}{15}\left(4\mu_2\Omega_0^2 + 5\mu_3\right)\tau + \mathcal{O}\left((\frac{4\tau\mu_3}{3})^2\right)\right) \quad \text{(A.40)}$$

Note that $V = -\frac{\gamma\sqrt{Z}}{24}W$, therefore the unperturbated dimensionless amplitude Ω_0^2 is linearly proportional to the external voltage input V_{inp}

$$\Omega_0^2 = 24V_{inp}/(Z^{1/2}\left|\gamma\right|) \quad \text{(A.41)}$$

It is worth mentioning that eqn (A.39), (A.40) and (A.41) describe the evolution of one soliton in the presence of a perturbation characterized by the amplitudes $M_1\left(\tau\right)$ and $M_2\left(\tau\right)$ which are expected to yield a slow change on the soliton parameters.[9] Therefore, the expansions appearing in eqn (A.39) and (A.40) provide a good estimation of the characteristic soliton travel time (in seconds) $T_0^{soliton} = \frac{360\alpha}{2\left(4\mu_2\Omega_0^2+5\mu_3\right)}$. This time should be, in principle, larger than the characteristic ion flow time, such that $T_0^{soliton} \gtrsim T_0^{G-actin} = 2\pi C_o\sqrt{Z^2 - \left(R_l + R_t\right)^2}$. Additionally, for the electrolyte conditions and models considered in the present article, we have $\Omega_0^2 \lesssim 1$, $L/Z \ll 1$ and $R_t/R_l \ll 1$ which yield the following approximate implicit equation for the impedance

$$\frac{360\alpha}{2\left(4\mu_2\Omega_0^2 + 5\mu_3\right)} \simeq \left(\frac{3ZC_o}{16}\frac{Z}{R_l}\right) \gtrsim 2\pi C_o\sqrt{Z^2 - \left(R_l + R_t\right)^2} \simeq 2\pi C_o\sqrt{Z^2 - R_l^2}$$

with solution $Z \gtrsim 25.1128R_l$ and $T_0^{soliton} \gtrsim 50.1858\pi C_o R_l$.

Appendix B. Numerical and Analytic Solution Comparison

We solve equation (8) numerically by using periodic boundary conditions $W\left(\xi,\tau\right) = W\left(-\xi,\tau\right)$ and a voltage input signal:

$$W\left(\xi,0\right) = -2\Omega_0^2 sech^2\left[\xi\right] \quad \text{(B.1)}$$

The artificially periodic boundary conditions were imposed to facilitate the resolution of the partial differential equation (8). However, for lengths of the microfilament big enough it does not affect the solution of the system.[13]. Equation (8) was solved using the commercial software Mathematica 11.0.[2] We applied the numerical method of lines algorithm, which is an efficient approach to numerically solve partial differential equations provided it is an initial value problem. This method discretizes all but one dimension, then integrates the semi-discrete problem as a system of Ordinary Differential Equations (ODEs) or Differential-Algebraic Equations (DAEs). Additionally, we configured some parameters to obtain the solution. We set the WorkingPrecision (e.g. how many digits of precision should be maintained in internal computations) to the MachinePrecision value (double-precision floating-point numbers: ≈ 16 decimal digits) . The AccuracyGoal and the PrecisionGoal (e.g. how many effective digits of accuracy and precision, respectively) were

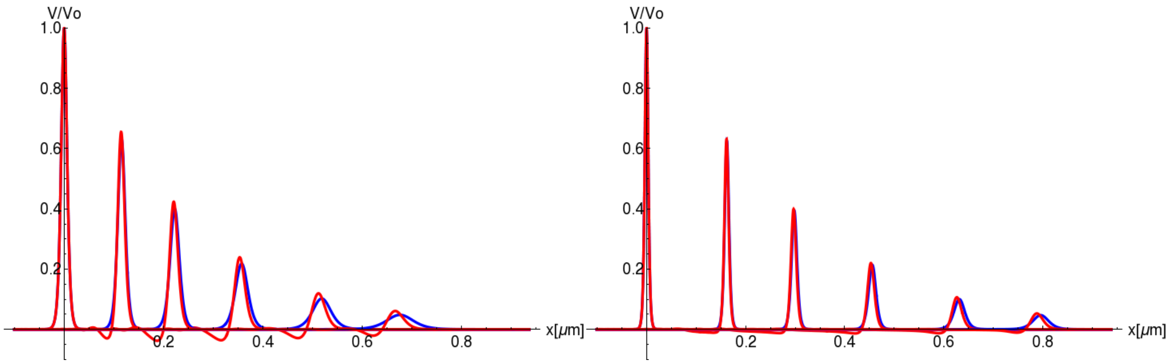

(a) Intracellular conditions.

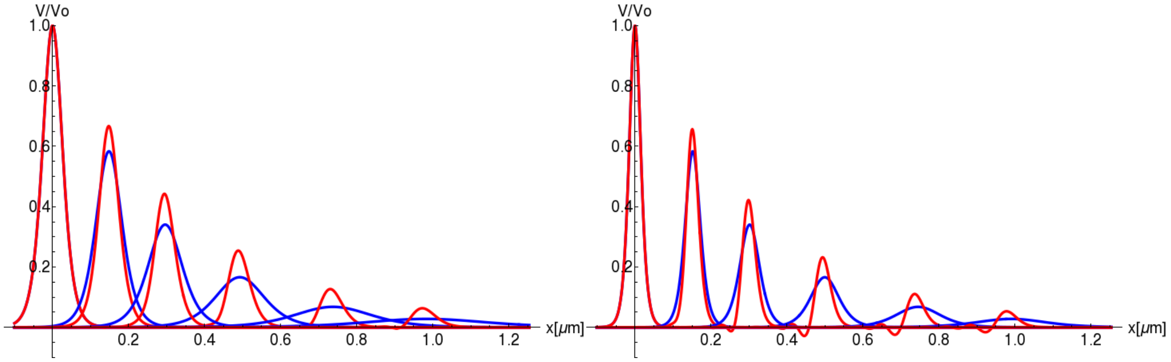

(b) In-vitro conditions

Figure B.2: Comparison between numerical (red color) and analytic (blue color) solutions. The figures to the left and right sides correspond to the electrical signal impulse amplitude generated by $0.05V$ and $0.15V$ input voltage peaks, respectively. The electrical impulse peaks correspond to the snapshots mentioned in Fig. 6

set to a value equal to half the setting for WorkingPrecision. The InterpolationOrder of the solution (e.g. continuity degree of the final output) was set to 6 for the ξ variable and 3 for the τ variable. The MaxStepFraction (e.g. maximum fraction of the total range to cover in a single step) was equal to 1/10, the MaxStepSize (e.g. maximum size of each step) was defined as the inverse of MaxStepFraction (10) and the MaxSteps (e.g. maximum number of steps to take in generating a result) was set to 10000. In the case of the NormFunction parameter, we used an infinity-norm.[2] It is worth mentioning that the inductance value considered in this work does not affect the numerical solution obtained for the soliton.

Fig. B.2 shows the soliton profile comparison between the numerical and the approximate analytic solution (9) for both intracellular and in-vitro conditions, obtaining a good visual matching over the whole domain. In general, there was a short and intermediate time evolution where the adiabatic approximation is valid. Certainly, at longer times the perturbation increases the impact on the soliton shape and tails. Overall, the peak position and width, as well as the kern velocity between the numerical and analytic solutions in intracellular conditions, are in very good agreement. Whereas, the analytic solution predicts a wider and more attenuated soliton for in-vitro conditions. Higher order approximations and multisoliton solutions will be considered in a future work.

References

[1] John Newman. *Electrochemical Systems*, chapter 1. Englewood Cliffs, N.J. Prentice-Hall, 1973.

[2] Wolfram Research, Inc. *Mathematica Version 11.0*, 2016.

[3] Yizhak Marcus. Ionic radii in aqueous solutions. *Chemical Reviews*, 88(8):1475–1498, 1988.

[4] David C. Grahame. The electrical double layer and the theory of electrocapillarity. *Chemical Reviews*, 41(3):441–501, 1947. PMID: 18895519.

[5] D. L. Sekulić, B. M. Satarić, J. A. Tuszyński, and M. V. Satarić. Nonlinear ionic pulses along microtubules. *The European Physical Journal E*, 34(5):49, 2011.

[6] S. Novikov, S.V. Manakov, L.P. Pitaevskii, and V.E. Zakharov. *Theory of Solitons: The Inverse Scattering Method*. Plenum, New York, 1984.

[7] M. Ablowitz and H. Segur. *Solitons and the Inverse Scattering Transform*. Society for Industrial and Applied Mathematics, 1981.

[8] V.I. Karpman and E.M. Maslov. A perturbation theory for the korteweg-de vries equation. *Physics Letters A*, 60(4):307 – 308, 1977.

[9] V. I. Karpman and E. M. Maslov. Perturbation theory for solitons. *Zh. Eksp. Teor. Fiz.*, 73:537–, August 1977.

[10] M. A. Allen and G. Rowlands. A solitary-wave solution to a perturbed kdv equation. *Journal of Plasma Physics*, 64(4):475–480, 2000.

[11] V I Karpman. Soliton evolution in the presence of perturbation. *Physica Scripta*, 20(3-4):462, 1979.

[12] E. M. Maslov. Perturbation theory for solitons in the second approximation. *Theoretical and Mathematical Physics*, 42:237–245, 1980.

[13] Jan Ole Skogestad and Henrik Kalisch. A boundary value problem for the kdv equation: Comparison of finite-difference and chebyshev methods. *Mathematics and Computers in Simulation*, 80(1):151 – 163, 2009. Nonlinear Waves: Computation and Theory {VII}.
